# Seroconversion to Seasonal Influenza Viruses after A(H1N1)pdm09 Virus Infection, Quebec, Canada

**DOI:** 10.3201/eid1807.111680

**Published:** 2012-07

**Authors:** Mariana Baz, Jesse Papenburg, Marie-Eve Hamelin, Manale Ouakki, Danuta M. Skowronski, Gaston De Serres, Guy Boivin

**Affiliations:** Author affiliations: Centre Hospitalier Universitaire de Quebec, Quebec City, Quebec, Canada (M. Baz, J. Papenburg, M.-E. Hamelin, G. Boivin);; Laval University, Quebec City (M. Baz, J. Papenburg, M.-E. Hamelin, G. Boivin);; Institut National de Santé Publique du Quebec, Quebec (M. Ouakki, G. De Serres);; British Columbia Centre for Disease Control, Vancouver, British Columbia, Canada (D.M. Skowronski)

**Keywords:** Cross-reactive immunity, seasonal influenza, pandemic influenza, microneutralization, viruses, Canada

## Abstract

We looked for cross-reactive antibodies in 122 persons with paired serum samples collected during the 2009 pandemic of influenza virus A(H1N1)pdm09. Eight (12%) of 67 persons with A(H1N1)pdm09 infection confirmed by reverse transcription PCR and/or serology also seroconverted to the seasonal A/Brisbane/59/2007 (H1N1) virus, compared with 1 (2%) of 55 A(H1N1)pdm09-negative persons (p<0.05).

The role of seasonal 2008–09 trivalent inactivated influenza vaccines in protecting against influenza A(H1N1)pdm09 virus remains controversial (*1*). Recent reports indicated that prior infections with seasonal influenza A viruses protected against A(H1N1)pdm09 virus infection, suggesting the presence of cross-reactive antibodies (*2*). Several studies have proposed that humoral immunity and conserved B- and T-cell epitopes contribute to heterosubtypic protection (*3*,*4*). Our objective was to determine whether A(H1N1)pdm09 infection induced cross-reactive antibodies against seasonal influenza A (H1N1) and A (H3N2) viruses.

## The Study

This investigation was part of a trial evaluating A(H1N1)pdm09 transmission among household contacts, conducted during the first wave of the 2009 pandemic (May–July 2009) in Quebec City, Quebec, Canada (*5*). Clinical data and samples were serially obtained from index case-patients and their contacts in 42 households. Nasopharyngeal secretions were collected from all participants during the first visit and tested by 2 different assays: a conventional reverse transcription PCR (RT-PCR) targeting the hemagglutinin gene of A(H1N1)pdm09 virus (*6*) and a universal RT-PCR targeting the matrix gene of all influenza A viruses (*7*). Blood was collected from persons >7 years of age at their initial visit (acute-phase sample) and 3–4 weeks later (convalescent-phase sample). Serum was tested by microneutralization assay according to World Health Organization standard protocols with minor modifications (*8*).

This serologic study comprised 122 persons from the 42 households. Twenty-four persons were RT-PCR–confirmed index case-patients (median age 15 years, range 7–56 years), and 98 were household contacts (median age 30.5 years, range 7–61 years), of whom 34 also were positive for A(H1N1)pdm09 virus by RT-PCR. For 67 patients (median age 20 years, range 7–61 years), A(H1N1)pdm09 was confirmed by RT-PCR and/or microneutralization assay: 10 (15%) by RT-PCR alone, 9 (13%) by microneutralization assay alone, and 48 (72%) by RT-PCR and microneutralization assay. Of the 67 A(H1N1)pdm09-infected persons, 8 (12%) seroconverted to A/Brisbane/59/2007 (A[H1N1] vaccine strain for 2008–09) (Table A1). Seven A/Brisbane/59/2007 seroconverters were RT-PCR positive and A(H1N1)pdm09 seroconverters, and 1 was RT-PCR positive and a A(H1N1)pdm09 nonseroconverter. In comparison, 1 (2%) of the 55 A(H1N1)pdm09-negative patients seroconverted to A/Brisbane/59/2007 (Fisher exact test, p<0.05). Seasonal influenza viruses were not circulating in the province of Quebec at the time of this study. Only 1 of 9 A/Brisbane/59/2007 seroconverters had previously received the inactivated 2008–09 seasonal influenza vaccines. No participants were vaccinated against A(H1N1)pdm09 virus, and none received antiviral therapy or prophylaxis.

We then assessed whether this cross-reactivity was limited to the A/Brisbane/59/2007 strain, the most recent seasonal A (H1N1) virus to have circulated before A(H1N1)pdm09 virus, or whether it was broader. To this end, we tested all paired serum samples against an older seasonal A (H1N1) influenza virus, i.e., A/New Caledonia/20/1999 (H1N1 vaccine strain used during the 2000–01 through 2006–07 seasons), and a past A(H3N2) virus, i.e., A/Panama/7/2004 (H3N2 vaccine component used during the 2000–01 through 2003–04 seasons). Seven (10%) A(H1N1)pdm09 virus–positive persons also seroconverted to A/New Caledonia/20/1999(H1N1), all of whom were RT-PCR-positive and A(H1N1)pdm09 virus seroconverters, whereas none of the A(H1N1)pdm09 virus–negative persons seroconverted to this older strain (p<0.05). On the other hand, seroconversion rates for A/Panama/7/2004(H3N2) did not differ significantly between A(H1N1)pdm09 virus-positive (9%) and -negative (5%) patients. In addition, we identified 4 (6%) persons with laboratory-confirmed A(H1N1)pdm09 virus infections who seroconverted to both seasonal (H1N1) viruses and 2 (3%) who seroconverted to A/Brisbane/59/2007(H1N1) and A/Panama/2007/99(H3N2) (Table). Participant 44C, a household contact of a confirmed case-patient with a negative RT-PCR for A(H1N1)pdm09 and low antibody titers in the convalescent-phase serum, showed cross-neutralizing antibodies meeting 4-fold seroconversion criteria for A/Brisbane/59/2007(H1N1) and A/Panama/7/2004(H3N2).

**Table:** Clinical features and microneutralization antibody titers against influenza A(H1N1)pdm09 and seasonal influenza A viruses of persons who seroconverted to >2 influenza viruses, Quebec City, Quebec, Canada, 2009*

Participant no./age, y	RT-PCR for A(H1N1) pdm09				Titers, acute phase/convalescent phase (-fold increase)			
		Symptoms			A/Quebec/147023/ 2009; A(H1N1)pdm09	A/Brisbane/59/ 2007 (H1N1)	A/New Caledonia/ 99 (H1N1)	A/Panama/2007/99 (H3N2)
		ARI	ILI					
39A/7	+	+	+		<10/160 (16)	<10/320 (32)	<10/160 (16)	320/160 (0)
39C/11	+	+	+		<10/40 (4)	10/160 (16)	<10/40 (4)	2,560/2,560 (0)
49A/28	+	+	+		<10/160 (16)	40/160 (4)	<10/20 (2)	320/320 (0)
49B/23	+	+	+		<10/640 (64)	<10/160 (16)	40/160 (4)	40/40 (0)
55F/7	+	+	+		<10/40 (4)	10/40 (4)	20/80 (4)	10/10 (0)
16C/7	+	+	+		<10/80 (8)	160/320 (2)	40/160 (4)	80/80 (0)
56A/12	+	+	+		<10/40 (4)	80/320 (4)	40/80 (2)	2,560/2,560 (0)
65B/40	+	+	+		<10/320 (32)	<10/20 (2)	10/40 (4)	320/320 (0)
03E/17	+	+	+		<10/80 (8)	40/80 (2)	160/160 (0)	640/5120 (8)
10B/14	+	+	+		<10/80 (8)	20/20 (0)	80/320 (4)	640/640 (0)
11C/61†	+	+	+		<10/1,280 (128)	40/160 (4)	160/160 (0)	320/1,280 (4)
44B/9	+	+	+		<10/<80 (8)	1,280/640 (0)	1,280/1,280 (0)	160/640 (4)
44C/44	–	+	–		<10/20 (2)	10/80 (8)	80/80 (0)	160/640 (4)
58A/13	+	+	+		<10/80 (8)	<10/20 (2)	10/20 (2)	1,280/5,120 (4)
58B/43	+	+	–		<10/160 (16)	<10/10 (0)	<10/<10 (0)	20/160 (8)

*RT-PCR, reverse transcription PCR; ARI, acute respiratory illness (i.e., presence of ≥2 of the following signs/symptoms: fever [≥37.8°C] or feverishness, cough, sore throat, or rhinorrhea); ILI, influenza-like illness (i.e., fever and cough and/or sore throat); +, positive; –, negative. †Received seasonal vaccine in 2008–09.

## Conclusions

During this study, the only influenza virus detected in the province of Quebec was A(H1N1)pdm09 virus. Yet, 8 (12%) of 67 A(H1N1)pdm09 virus–infected persons in our study had a concomitant significant increase in microneutralization antibody titers against the most recent A/Brisbane/59/2007(H1N1) strain, of whom 5 persons had 4–8-fold, 2 had 16-fold, and 1 had 32-fold rises. In addition, 4 of these 8 persons also seroconverted to an older A/New Caledonia/20/1999(H1N1) virus, of whom 3 persons had 4-fold and 1 had 16-fold rises between acute-phase and convalescent-phase serum. The cross-reactivity observed in the study population does not seem to be completely subtype specific because some persons also showed rising titers against an old influenza A (H3N2) strain (A/Panama/2007/99), although in this case, seroconversion rates did not differ significantly between A(H1N1)pdm09 virus–positive and –negative persons.

A recent study in Hong Kong of 28 paired serum samples showed that infection with the pandemic virus could broaden cross-reactive immunity to other recent subtype H1 swine viruses. In contrast to our study, perhaps because of the small number of participants or older age of A(H1N1)pdm09 virus–positive case-patients (30.5 vs. 20 years), no cross-reactive response was shown against the more recent seasonal influenza virus A/HK/400599/2008(H1N1) (*9*).

We could not determine the extent to which past seasonal influenza vaccinations and/or natural infections contributed to the generation of cross-neutralizing antibodies to A(H1N1)pdm09 and the seasonal influenza strains. Our next step will be to investigate potential cross-neutralizing determinants between these seasonal and pandemic viruses. Neutralizing antibodies that bind to the stalk region of HA2 have been shown to confer broad cross-neutralizing activity against several subtypes of viruses across clades and to provide protection in animal models (*10*,*11*). Six of the 8 persons who seroconverted to A/Brisbane/59/2007(H1N1) by microneutralization assay did not meet the 4-fold criteria by hemagglutinin inhibition assay (data not shown), suggesting that the cross-reactivity might result from conserved epitopes in the stalk region of HA2 or in other proteins. Greenbaum et al. recently showed that, overall, 49% of the epitopes reported in recently circulating seasonal A (H1N1) strains were conserved in the A(H1N1)pdm09 virus (*12*). Specifically, 31%, 41%, and 69% of the B-cell, CD4^+^ and CD8^+^ T-cell epitopes, respectively, were conserved. Natural infection with A(H1N1)pdm09 virus also could have elicited cross-reactive responses against internal components of older viral strains (*13*). We find intriguing the elaboration of cross-reactive neutralizing antibodies to more recently circulating influenza A (H1N1) strains as a result of novel A(H1N1)pdm09 virus infection, whereas the reverse has not generally been evident in serosurveys for cross-reactive A(H1N1)pdm09 antibody, except in elderly persons who had substantial cross-reactive antibodies to A(H1N1)pdm09 virus (*14*,*15*). Unfortunately, because of the small sample size of our study and lack of serum from children <7 years of age, we could not assess whether cross-reactivity was an age-dependent phenomenon. However, all but 3 of the cross-reactive seroconverters (13/16 [81%]) were 7–30 years of age. To explore preferential responses to the original infecting virus (original antigenic sin), we assessed cross-reactivity for the older A/New Caledonia/99(H1N1) virus that was potentially the priming antigen for some of our younger participants or was closely related to the priming antigen in older participants. However, seroconversion rates for A/New Caledonia (10%) were comparable to those of the more recent A/Brisbane/59/2007(H1N1) strain (12%), and thus we could not distinguish original antigenic sin on that basis. These antigens may have been too closely related antigenically to demonstrate that in this young cohort.

Our work supports the notion that natural A(H1N1)pdm09 virus infection induces broad heterosubtypic (H1 and even H3) responses. It also highlights the need for further investigation of the mechanisms behind cross-protection because they could be keys to creating improved influenza vaccines with broader protection.

## Appendix

**Table A1:** Seroconversion rates and GMTs for pandemic and seasonal influenza viruses, Quebec City, Quebec, Canada, 2009*

A(H1N1)pdm09 virus	Age, y		Received seasonal vaccine in 2008–09	A/Quebec/147023/2009; A(H1N1)pdm09			A/Brisbane/59/2007 (H1N1)			A/New Caledonia/99 (H1N1)			A/Panama/2007/99 (H3N2)	
	Mean†	Median (range)		Seroconversion rate†	GMT (95% CI)		Seroconversion rate†	GMT (95% CI)		Seroconversion rate†	GMT (95% CI)		Seroconversion rate	GMT (95% CI)
Positive, n = 67‡	25.7	20 (7–61)	19 (28)	57 (85)	69.2 (49.9–95.9)		8 (12)	104.7 (73.5–149.1)		7 (10)	96.5 (65.5–142.4)		6 (9)	285.6 (188.5–432.6)
Negative, n = 55	31.2	35.5 (8–55)	13 (24)	0	5.9 (5.2–6.8)		1 (2)	41.0 (28.4–59.2)		0	34.4 (22.2–53.3)		3 (5)	124.4 (77.5–199.6)

*Values are no. (%) except as indicated. Seroconversion defined as an acute-phase serum titer of <10 with a convalescent-phase titer of >40 or a significant increase (>4-fold) in antibody titers between acute- and convalescent-phase serum samples. GMT, geometric mean titer;  †p<0.05. ‡A(H1N1)pdm09-positive persons were defined by positive reverse transcription PCR and/or seroconversion by microneutralization assay.
